# Somatic Embryogenesis from Mature Embryos of *Olea europaea* L. cv. ‘Galega Vulgar’ and Long-Term Management of Calli Morphogenic Capacity

**DOI:** 10.3390/plants9060758

**Published:** 2020-06-17

**Authors:** Rita Pires, Hélia Cardoso, Augusto Ribeiro, Augusto Peixe, António Cordeiro

**Affiliations:** 1 IIFA—Instituto de Investigação e Formação Avançada, Universidade de Évora, Pólo da Mitra, Ap. 94, 7006-554 Évora, Portugal; rnpires@uevora.pt; 2 MED—Mediterranean Institute for Agriculture, Environment and Development, Instituto de Investigação e Formação Avançada, Universidade de Évora, Pólo da Mitra, Ap. 94, 7006-554 Évora, Portugal; 3 DespertaFolia Lda., Universidade de Évora, Pólo da Mitra, Ap. 94, 7006-554 Évora, Portugal; viriatoribeiro@despertafolia.pt; 4 MED—Mediterranean Institute for Agriculture, Environment and Development, Departamento de Fitotecnia, Escola de Ciências e Tecnologia, Universidade de Évora, Pólo da Mitra, Ap. 94, 7006-554 Évora, Portugal; 5 INIAV—Instituto Nacional de Investigação Agrária e Veterinária, I.P., UEIS Biotecnologia e Recursos Genéticos, Estrada de Gil Vaz, Apartado 6, 7350-901 Elvas, Portugal; antonio.cordeiro@iniav.pt

**Keywords:** cyclic embryogenesis, olive, zygotic embryos

## Abstract

Several olive cultivars, characterized by high-quality olive oil show agronomical issues such as excessive vigor, high susceptibility to biotic and abiotic stresses, and low propagation ability. They are strong candidates for breeding based on new technologies to improve their performance in a short period of time. For this reason, the first step is developing efficient somatic embryogenesis (SE) protocols. Somatic embryogenesis in olive is highly genotype-dependent for both adult tissues and mature embryos as initial explants, requiring the development of specific protocols for each genotype. Trials using cotyledons and radicles as initial explants, isolated from ripe seeds from the Portuguese olive cv. ‘Galega vulgar’, gave more than 95% calli development. Radicles proved to be the most responsive tissue for SE induction, with an average of 2 embryos per callus after callus transfer to expression medium, and 14 embryos per callus after subculture on the olive cyclic embryogenesis medium (ECO). Embryogenic competence could be recovered after several subcultures on ECO medium that maintained cyclic embryogenesis for an indeterminate period of time. Embryo conversion and plant acclimatization were also attained with high success rates. Media management for cyclic embryogenesis maintenance is of general importance for SE protocols in any olive genotype. Somatic embryogenesis was thus attained for the first time in embryo-derived explants of cv. ‘Galega vulgar’.

## 1. Introduction

Olive trees are one of the oldest and most economically relevant fruit trees and oilseed crops in the Mediterranean region, being most of the cultivars used for olive oil production [1]. Two olive forms can be found in the Mediterranean basin, the wild olive (*Olea europaea* L. ssp. *europaea* var. *sylvestris*) and the cultivated olive (*Olea europaea* L. ssp *europaea* var. *sativa*), and, in many regions, the two forms coexist and are compatible [2,3]. There are currently more than 2000 olive cultivars and this genetic diversity is the main factor contributing to the olive oil singularity of each country and region [4,5]. Besides the organoleptic characteristics of fruits and oils produced, genetic diversity also represents a source of genetic information that could be further explored in breeding programs focused on specific agronomical traits [6]. Development of new cultivars must be focused on solving issues such as modification of growth habit, development of self-fertile plants to increase yield, development of totally self-sterile plants for conventional breeding, increasing fruit oil content and quality, increasing abiotic stress tolerance, increasing disease and pest tolerance, and production of plants with a higher adventitious rooting ability [7,8].

Olive transcriptomics, proteomics, and metabolomics [9,10,11,12] have begun to provide indications of candidate genes that may be key to the definition of an interesting phenotype. However, data from these high throughput approaches only has physiological value if the functionality is confirmed, usually by knockout or overexpression of target genes [13,14,15]. Functional validation requires the establishment of in vitro protocols for plant regeneration, where cells integrating the foreign DNA can regenerate an entirely new plant. Of the different strategies that could be followed for in vitro regeneration of a new plant, somatic embryogenesis (SE) is the most suitable tool for genetic transformation protocols. Somatic embryogenesis is recognized as an efficient morphogenic response upon external stress stimulus based on molecular and metabolic cell reprogramming that covers typically phases of dedifferentiation and de novo-differentiation [16]. The selection of this regeneration system is mainly based on high proliferation rates [17,18,19,20] and frequent single-cell origin of the differentiated somatic embryos, which avoids the problem of chimeras [8,19,21,22].

Attempts to induce SE in olive started in the 1980s when Rugini and Tarini [23] used seedling-derived roots to induce somatic embryos. Later, the first successful protocols were achieved by Rugini [24] for cultivars ‘Frantoio’, ‘Moraiolo’, ‘Leccino’, and ‘Dolce Agogia’. Further research, mainly based on juvenile tissues of immature and mature zygotic embryos or petioles taken from in vitro seedlings as initial explants [25,26,27,28,29,30,31] allowed the establishment of some effective protocols (see Cardoso et al. [32] and [33] for details). Recalcitrance is associated with the use of adult plant tissues with exceptions known for cvs. ‘Canino’, ‘Moraiolo’, ‘Chetoui’,‘Dahbia’, and ‘Picual’, for which were used petioles/leaf tissues taken from in vitro growing plants, to successfully induce SE and further embryos differentiation [34,35,36,37]. Embryos conversion, known as a key step in an efficient SE protocol, was limited to cv. ‘Canino’ and ‘Moraiolo’ [34]. Successful reports have also been described in some wild olive genotypes. The first report is known from Capelo and co-authors [38], which demonstrated the possibility to use petioles/leaf tissues taken from plants established under greenhouse conditions (no juvenile or rejuvenated material), to induce SE response with efficient differentiation of somatic embryos. More recently, Narváez and co-authors [39] described a protocol focused on different olive wild genotypes differing in their response to defoliating *Verticillium dahlia*, in which was stated the use of shoot apex collected from in vitro plantlets as the most appropriated tissue to induce SE and further efficient embryos conversion.

The efficiency of SE induction depends on several factors with a complex interaction among them. The main factors affecting SE efficiency are the genotype of the donor plant, the type of explant taken for the establishment of in vitro cultures, the development and physiological stage, the growth conditions (mainly photoperiod and temperature), and the chemical composition of the culture media (including type and concentration of growth regulators) [40,41]. The genotype strongly affects the ability of a tissue to dedifferentiate and de novo acquire meristematic competence and to differentiate embryogenic structures [39,42,43,44]. Individual genotypes within the same species vary greatly in embryogenic capacity, reflecting substantial differences in the ability to activate key elements for the achievement of embryogenic competence [41] which shows the need to develop improved methodologies for each genotype. Results reported by Narváez and co-authors [39] emphasize this dependence, showing SE induction efficiency in two olive wild genotypes from the four initially considered, and embryos conversion from a single one.

Besides the genotype, the developmental stage and age of the explants used as start material, are key factors determining the success of SE induction. Several reports on woody plant species, *Vitis vinifera* [40,41] *Picea abies* [45], *Prunus incisa* [46], *Pinus radiata* [47], and *Eucalyptus globulus* [48], emphasize the effect of explant developmental stage on embryogenesis efficiency.

Concerning the culture media formulations, the OM (Olive medium) [49] medium with 1 g L^−1^ casein hydrolysate and devoid of glutamine or the MS (Murashige and Skoog) [50] basal salts, are generally used in olive SE for both, juvenile and adult initial explants. High auxin/cytokinin ratios provided the best results for the culture induction phase [25,27], while culture media lacking growth regulators, or with a low auxin concentration, were used for embryo differentiation and development [28,35,51,52].

Somatic embryogenesis has not been routinely and widely used in the propagation of *Olea* spp. where few cultivars have been used to collect plant material to induce SE response (see review in Cardoso et al. [32] and Sánchez-Romero [33]). The use of mature zygotic embryos as initial explants to attain this goal is far from optimal, but the information acquired from those trials is usually fundamental to obtain results when adult explants of a selected genotype are used. The cv. ‘Galega vulgar’ chosen for this study is recalcitrant for adventitious root formation when semi-hardwood cuttings are used, and previous work developed by our research group has been focused on understanding the mechanisms underlying that morphogenic process by following different omic approaches [53,54]. Data achieved from the different omic platforms need functional validation as a step forward on this research topic, which required prior development of an effective protocol for SE. The present research reveals the establishment of an efficient protocol that will make available plant material to be used in genetic transformation and gene editing of olive varieties.

## 2. Results and Discussion

### 2.1. Induction of Somatic Embryogenesis

Data on callus formation and morphology were collected 21 days after inoculation. The summary of the variance analysis presented in Table 1 shows that only the initial explant type significantly affected the callus development rate.

**Figure 1:**
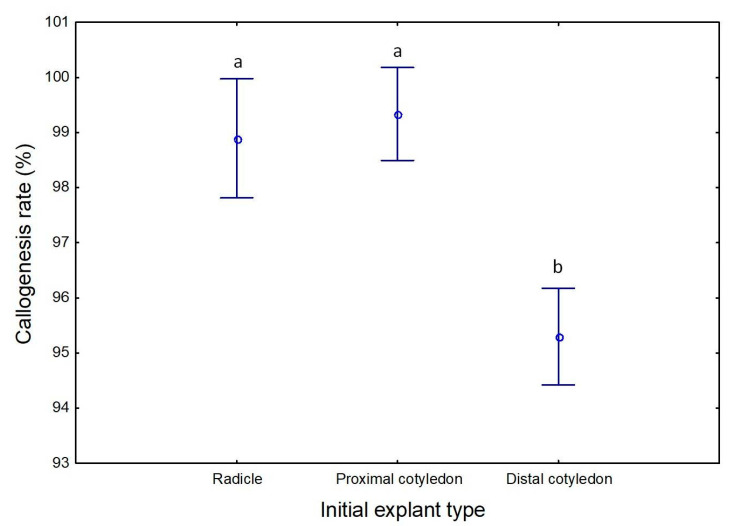
Callus formation rate on induction media, according to the initial explant type, radicle, proximal, and distal region of cotyledons. Different letters correspond to statistically significant differences (*p* ≤ 0.05).

Although all the explants presented a high rate of callogenesis, explants from the distal region of cotyledons with an average of 95.3%, was significantly lower than that observed for radicles (98.9%) and the proximal region of cotyledons (99.3%) (Figure 1). High levels of callogenesis were also reported by Orinos and Mitrakos [55] and Rugini and Silvestri [56] working with olive zygotic embryos *of Olea europaea* var. *sylvestris* and var. *sativa*, respectively. Different callogenesis rates caused by the use of different olive embryo regions were also previously reported by Orinos and Mitrakos [55] and Mitrakos et al. [25] who obtained the best results when radicles were used as initial explants.

**Figure 2:**
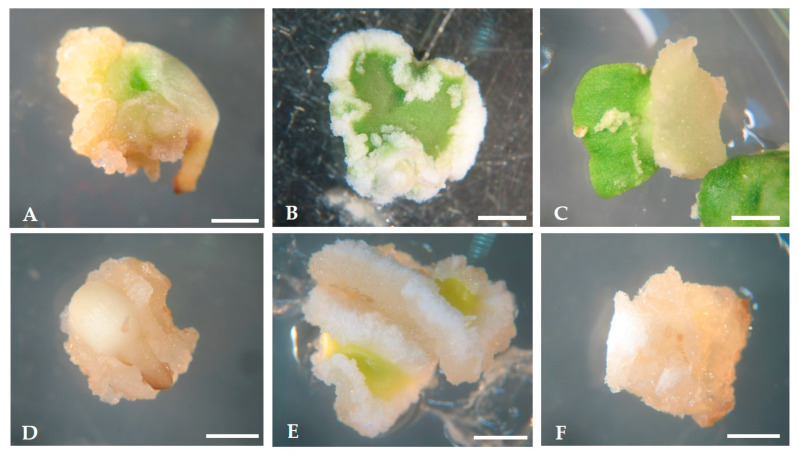
Calli formation in the two light regimes, 16 h photoperiod (**A**–**C**) and darkness (**D**–**F**), for the three types of initial explants, radicles (**A**,**D**), proximal cotyledon region (**B**,**E**) and distal cotyledon region (**C**,**F**). Bars: 10 mm.

In the case of cultures maintained under 16 h photoperiod, a volume increase was followed by a color change from white to green due to chlorophyll production by chloroplast activity. After 21 days in culture, the development of calli was visible, mainly at the explant peripheral and wounded regions. Similar evolution patterns were reported by Mazri et al. [57], working with cv. ‘Dahbia’. The developed calli presented a friable structure and color from light yellow to white (Figure 2). No differences were observed on the calli developed under light (Figure 2A–C), or dark conditions (Figure 2D–F).

**Figure 3:**
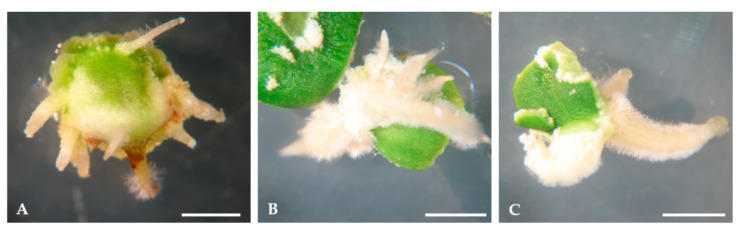
Neo-formation of roots during the induction phase. (**A**) Evolution from radicles, (**B**) from the proximal region of cotyledons, and (**C**) from the distal region of cotyledons. Bars: 5 mm.

In addition to calli development, the neo-formation of roots was observed during the induction phase (Figure 3). Root neo-formation was higher on calli developed from radicles and proximal region of cotyledons (data not shown).

### 2.2. Expression of Somatic Embryogenesis

All the explants that developed calli during the induction phase were transferred, as suggested by Orinos and Mitrakos [55], to the culture medium with the same basal composition (OMc medium) but devoid of growth regulators. The two light regimes, 16 h and 0 h of light were maintained in this phase.

**Figure 4:**
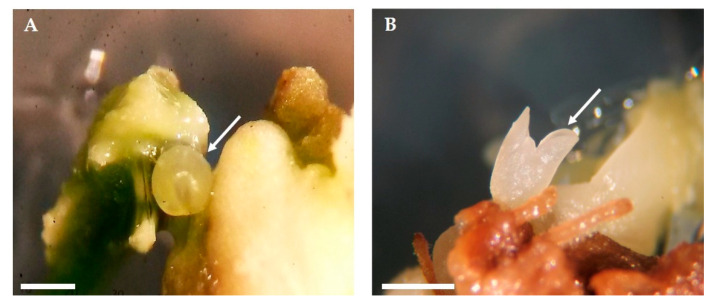
Somatic embryos on the two light regimes 30 days after inoculation in OMc medium devoid of growth regulators. Somatic embryo at the globular stage arising in calli established under 16 h photoperiod (**A**) and embryo at the cotyledonary stage arising in calli established under dark condition (**B**). Bars: 10 mm.

Thirty days after calli transfer, the first embryogenic structures differentiated at the surface of embryogenic calli (Figure 4). Somatic embryos were visible on calli arising under both light conditions, 16 h photoperiod (Figure 4A), and darkness (Figure 4B). Embryogenic calli present a different structure from the calli initially developed in the induction medium, exhibiting a granular appearance with milky white color.

In general, the percentage of embryogenic calli was low (7 to 22%), as well as the average of differentiated embryos per responsive calli (1.25 to 2.55 embryos), with the best results achieved in calli developed from radicles established under 16 h photoperiod (Table 2).

### 2.3. Management of Embryogenic Potential-Cyclic Embryogenesis Induction and Maintenance

Aiming to increase the efficiency of the protocol on the capacity to differentiate somatic embryos, previously induced embryogenic calli were sub-cultivated into fresh OMc medium for an additional 30 days under the same culture conditions. Considering the results achieved during the first sub-culture, only the calli developed from radicles were selected for this second sub-culture. Observations performed after 30 days showed that the morphogenic response, contrary to expectations, did not increase, and, in addition, embryogenic calli started losing growth capacity and became necrotic (data not shown).

Trying to recover the embryogenic capacity, all calli still showing a healthy appearance were sub-cultivated into Olive Cyclic Embryogenesis (ECO) medium. The ECO medium was proposed by Pérez-Barranco et al. [51] as being efficient for cyclic embryogenesis in olive. Thirty days after this transfer, the calli growth capacity was restored and new somatic embryos differentiated. The number of embryos per explant increased from 2.54 on OMc medium to 7.5 on the first sub-culture in ECO medium and to 13.6 in the second sub-culture, achieved with calli developing under 16 h photoperiod (Table 3).

The higher number of embryos was again achieved under light, the results being significantly different than those obtained in the dark. This result disagrees with the results reported by several authors that reported darkness as an essential condition for somatic embryo achievement in *Olea* spp. [24,28,34]. In other species, *Cydonia oblonga* [58], *Castanea sativa* [59] *Coffea canephora* [60], *Quercus suber* [61], and *Phalaenopsis* [62], the light seems to be essential to induce SE. Based on these results, the remaining trials were performed only under 16 h light.

**Figure 5:**
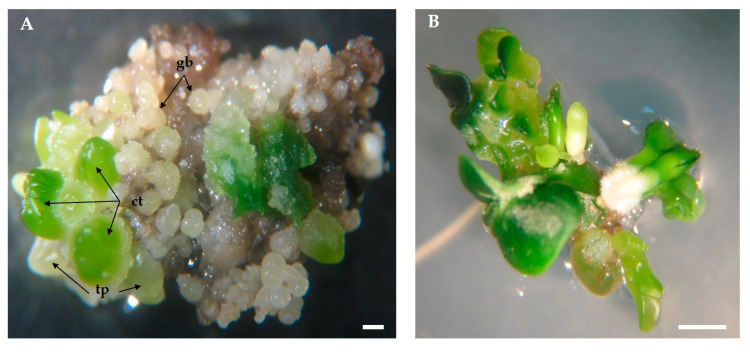
Embryogenic calli from radicle in ECO medium under 16 h photoperiod. (**A**) After first sub-culture with the identification of somatic embryos at different developmental stages (gb: globular, tp: torpedo, ct: cotyledonary), (**B**) After the 3rd sub-culture with secondary embryogenesis visible. Bars: 10 mm.

Embryos at different developmental stages, from globular to cotyledonous, were simultaneously identified on the same callus (Figure 5A). This asynchronous development has been commonly found in SE across plant species [41,63,64]. Higher homogeneity on embryo development can only be achieved with the selection of embryogenic cell lines, as proposed by Frederico et al. [65].

The ability of cultures to proliferate indefinitely through cyclic embryogenesis is one of the most important aspects of SE [44]. However, a progressive decline of embryogenic calli growth and a loss of their morphogenic potential in cultures maintained through repetitive sub-cultivation has been reported by Negrutiu and Jacobs (1978), Fridborg and Eriksson (1975), and Molle et al. (1993) cited in Bhojwani and Razdan [66]. In olive, Bradaï et al. [67] recently reported that cultures maintained by repetitive sub-cultivation over prolonged periods can maintain high rates of embryogenic calli growth but gradually lose the capacity to differentiate embryos. In a review about somatic embryogenesis in *Olea* spp., Sánchez-Romero [33], highlighted the involvement of different factors (e.g., initial explant and culture media composition) on calli proliferation and long-term maintenance of morphogenic capacity. Also, the potential of the ECO medium to support the long-term proliferation of olive embryogenic calli and its morphogenic capacity has been emphasized [33,67], but no information is available about the number of sub-cultures in which the calli are capable of maintaining their highest embryogenic potential neither if this response can be recovered from cultures starting losing it while growing in other culture media, as observed by Cerezo et al. 2011 [30].

Different causes have been proposed for the decline of morphogenic potential during the maintenance of embryogenic cultures. Prolonged upkeep in proliferation media with added growth regulators, is one cause that has also been associated with the appearance of somaclonal variations, easily detected by changes in morphological characteristics of regenerated plants when compared with the donor plant [62,68,69]. This behavior is probably the result of genetic and/or epigenetic variations in the cell population that consequently may affect the integrity of the previously acquired developmental pathway [68,70,71].

**Figure 6:**
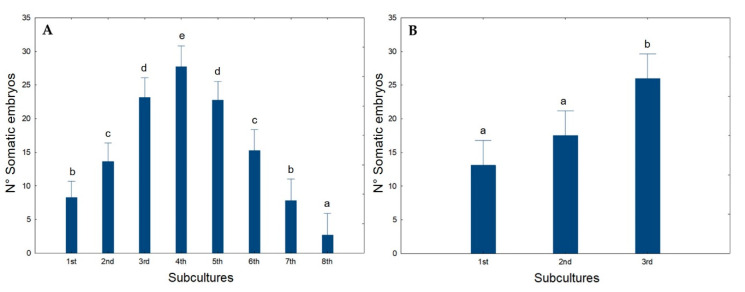
(**A**) Average number of somatic embryos per calli achieved during 8 sub-cultures in the ECO medium. (**B**) Average number of somatic embryos per calli obtained in the first 3 sub-cultures after reinoculation in the ECO medium. Different letters correspond to statistically significant differences (*p* ≤ 0.05).

To evaluate the capacity of the ECO medium to preserve long-term cyclic embryogenesis in olive, a sequence of eight sub-cultures was initiated, with calli transfer every 30 days to fresh ECO medium. The results are given in Figure 6.

The number of somatic embryos per explant increased until the 4th sub-culture. This increase was mainly due to the development of new somatic embryos from previous embryogenic structures already present on the calli sub-cultivated, i.e., secondary embryogenesis (Figure 5B). However, after the 4th sub-culture, although no changes were visible in calli appearance, the morphogenetic capacity started decreasing, and, by the 8th sub-culture, the values are below those achieved in the first sub-culture (Figure 6A). Secondary SE has been reported in the past in olive, e.g., by Rugini and Caricato [34] and by Benelli et al. [72] which highlighted it as an efficient form to maintain olive embryogenic cultures and somatic embryo production.

To recover the SE potentiality, explants were transferred back to the initial induction medium (OMc medium) for a month. After that, they were reinoculated into ECO medium and three sub-cultures were performed at 30 days intervals. From Figure 6B, calli recovered morphogenic capacity during the first subculture, acquiring levels of embryo formation similar to those previously obtained. This strategy can be used to maintain the cyclic embryogenesis in olive somatic embryogenic calli for an indeterminate period of time.

### 2.4. Embryo Conversion and Plant Acclimatization

**Figure 7:**
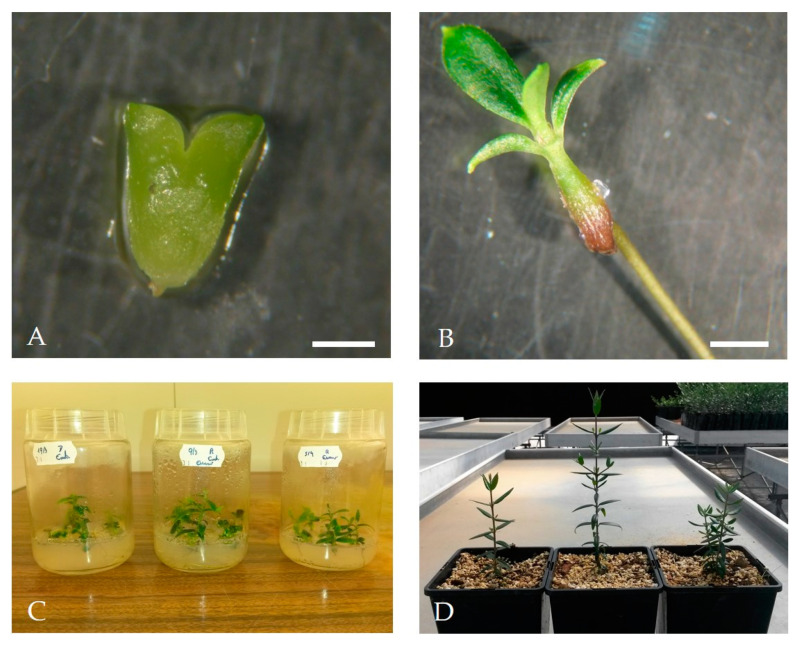
(**A**) Somatic embryo at the cotyledonary stage in light condition; (**B**) Plant from the somatic embryo in OMc without growth regulators; (**C**) Embryos convert into normal plants after 12 weeks and (**D**) Plants after acclimatization stage. Bars: 10 mm.

To induce somatic embryo conversion into plants, embryos at the cotyledon stage larger than 3 mm (Figure 7A) were individualized and inoculated into Petri dishes containing OMc medium without growth regulators for 6 weeks. After this period, embryos were transferred to flasks containing culture medium with the same composition for another 6 weeks in a plant growth chamber at 24 °C/22 °C day/night with 95–100 μmol m^−2^ s^−1^ light intensity (Figure 7B).

After the 12 week period on OMc medium, embryos converted into normal plants, with well-developed root and aerial systems (Figure 7C). From the total of isolated embryos, 60% efficiently converted into normal plants. Embryos conversion has been highlighted as a key point in the establishment of an efficient SE protocol in different plant species [32,73] including olive [74]. When tissues taken from zygotic embryos were used as initial explants to induce SE, high efficiency was achieved in the conversion process [26,28,30,31,55]. Leva et al. [26] and Rugini et al. [75] described the direct conversion of somatic embryos without the need of an intermediate stage of maturation. As mentioned by Sanchéz-Romero [33], the conversion of olive somatic embryos into plants did not meet rigorous maturation requirements. Even when individualized in earlier developmental stages, an acceptable conversion rate is achieved [76], unlike other species where somatic embryos only germinate when they reach advanced stages of development [39,77]. Recalcitrant behavior has been described in embryos conversion when mature olive plant tissues are used as initial explants, and exceptions are only known in cv. ‘Canino’ and ‘Moraiolo’ [34] and one wild olive genotype [39].

After the young plants reached about 10 cm, they were transferred to honeycomb trays for acclimation to ex vitro conditions as explained in material and methods. The substrate used consisted of a mixture of sand, perlite, and peat in the proportion 1:1:3 (v/v). Finally, the plants were transferred to 2 L pots containing a substrate with similar composition and transferred to a greenhouse (Figure 7D). At the end of this phase, it was possible to obtain an average rate of 75% acclimatized plants.

## 3. Materials and Methods

### 3.1. Plant Material

The fruits used for seed extraction were provided by Instituto Nacional de Investigação Agrária e Veterinária and were collected in Elvas, Portugal, in the Coleção Nacional de Referência de Cultivares de Oliveira, from 8-year-old trees of cv. ‘Galega vulgar’. The harvest was made at full ripeness, the pulp was removed manually, and after washing the seeds were kept in the cold (4 ± 1 °C) for 3 months to break the dormancy of the embryo. The endocarp was then broken with a manual press and the seeds isolated and placed in sterile water (autoclaved at 121 °C for 20 min) for 42 h in the dark at 24 ± 1 °C, before the surface disinfection process.

### 3.2. Seed Surface Disinfection and Explant Preparation

Seed disinfection consisted of the first wash with 70% (v/v) ethanol solution for 2 min followed by a wash with sterile bi-distilled water. Water was replaced by calcium hypochlorite solution (10% w/v) containing 0.1% Tween-20 (v/v), and the flasks were closed and kept under continuous agitation (~150 rpm) for 20 min. Finally, the solution was removed and seeds were rinsed three times with sterile bi-distilled water. In aseptic conditions, the radicles and cotyledons were excised from the interior of seeds and used as initial explants. The radicles were placed in culture as a whole while the cotyledons were separated and cut into distal and proximal portions before inoculation in culture media.

### 3.3. Embryogenesis Induction and Expression

For the embryogenesis induction phase, the explants were placed into Petri dishes (7 cm diameter) containing 25 ml of OMc culture medium [78] supplemented with 2.5 µM 6-dimethylallylamino-purine (2iP), 25 µM indole-3-butyric acid (IBA) [55] and jelled with 7 g L^−1^ Agar-Powder (VWR, Lisboa, Portugal). Cultures were maintained for 21 days at 25 ± 1 °C with a photoperiod of 16 h and a light intensity of 40–45 μmol m^−2^ s^−1^, or alternatively in the dark.

After the induction phase, explants were transferred to hormone-free OMc culture medium to promote the differentiation of the embryogenic structures (expression phase). Thirty days after inoculation explants were subcultivated on OMc culture medium, and cultures were kept under the same growth conditions as described above. Two subcultures were considered and for the second one, only calli from radicles were used.

### 3.4. Cyclic Embryogenesis

After two sub-cultures on expression medium, all embryogenic calli were transferred to olive ECO medium to induce cyclic embryogenesis [30,51,52,79]. The formulation of the ECO medium is based on the formulation of modified olive medium for SE (OMe) [78] containing ¼ OM macroelements, ¼ MS microelements, ½ OM vitamins, 50 mg L^−1^ myo-inositol, 20 g L^−1^ sucrose, 550 mg L^−1^ l-glutamine, and supplemented with 0.5 μM 2iP, 0.44 μM 6-benzyladenine (BA), 0.23 μM IBA, 1 g L^−1^ casein hydrolysate and 0.42 mM cefotaxime (sterilized by filtration as proposed by Rugini and Caricato [34]). The culture medium was jelled with 2.5 g L^−1^ Sigma-Phytagel.

Cultures were maintained during eight months by calli sub-culture into fresh culture medium every month. The culture conditions were initially maintained as previously described for the expression phase, but after the second sub-culture, only the 16 h photoperiod was maintained.

### 3.5. Recover of Calli Embryogenic Capacity

To recover embryogenic capacity, all calli maintained in ECO medium for eight sub-cultures were transferred to the induction medium previously described (OMc). Cultures were kept at 25 ± 1 °C with a photoperiod of 16 h and a light intensity of 40–45 μmol m^−2^ s^−1^. After 21 days, calli were transferred back to ECO medium where they remained for 3 sub-cultures.

### 3.6. Embryos Conversion and Plants Acclimatation

To promote embryos conversion, embryos larger than 3 mm were removed from the embryogenic calli following the procedure previously described by Bradaï et al. [69], and further cultivated in 180 mL glass bottles containing OMc medium devoid growth regulators. Cultures were kept at 25 ± 1 °C with a 16 h photoperiod and 40–45 μmol m^−2^ s^−1^ of light intensity.

Plantlets were maintained under in vitro conditions until the shoots reached about 10 cm in height. Young plants were then removed from the glass bottles and transferred to polypropylene honeycomb trays with 28 wells containing approximately 200 mL of substrate per well. The substrate consisted of a mixture of sand, perlite, and peat in the proportion of 1:1:3 (v/v).

To avoid dehydration, the trays were placed in stalls with a transparent plastic polyethylene cover to maintain high relative humidity. Plants were maintained under controlled conditions in a plant growth chamber with 24 °C /22 °C day/night temperature, 60% humidity, 16 h photoperiod and light intensity of 90 μmol m^−2^ s^−1^. After 15 days, the plastic cover was removed from the stalls and the plants remained for another 15 days under the same conditions.

Finally, the plants were transferred to 2 L pots containing a substrate with similar composition and transferred to a greenhouse.

### 3.7. Experimental Design and Statistical Analysis

For the experiments on the induction and expression of embryogenesis, three explant types (radicles, distal and proximal cotyledons) and two light conditions (16 h and 0 h photoperiod) were tested. The trial followed a complete factorial design, with each Petri dish having 10 explants acting as one replicate and with nine replications at least. Data on calli formation rates, development of adventitious structures, and the number of formed embryos were recorded.

For the experiments on cyclic embryogenesis, calli from radicles were transferred into ECO culture medium. Two photoperiod regimes were considered: 16 h and 0 h (dark). The trial also followed a complete factorial design, with 7 calli inoculated per Petri dish that acted as one replicate. The number of replicates varied for each culture condition tested, but at least 10 replications were used.

All data were tested for normality (by Shapiro-Wilk test) and submitted to analysis of variance (ANOVA) followed by Fisher’s (LSD) posthoc test. Significant differences were recorded for *p* ≤ 0.05. Values in percentage were transformed by arcsine of the square root before analysis.

## 4. Conclusions

Mature embryo structures (radicles and cotyledons) were extracted from fruits collected from trees of the Portuguese olive cv. ‘Galega vulgar’ and used to develop a protocol that enables plant regeneration via SE induction. Embryo induction, expression, and conversion were attained with high success rates. Cyclic embryogenesis, which is fundamental for use in transcriptomic/proteomic validation studies following a transgenic/gene editing approach was achieved. Information on embryogenic calli behavior during successive sub-cultures on cyclic embryogenesis culture media was obtained as well as a way to manage the morphogenic response of the calli, thus allowing embryo formation for an indeterminate period of time. This information on long-term maintenance of morphogenic calli is important for further proceed with functional validation studies that require the long-term availability of embryogenic material, able to efficiently convert transgenic plants.

## Figures and Tables

**Table 1:** Variance analysis summary for callogenesis rates during induction, as affected by the initial explant and light regime. Significant differences registered for values of *p* ≤ 0.05.

Variables	Sum of Squares	Degrees of freedom	Medium Square	F	*p*
Explant type	619	2	310	6.23	0.002
Photoperiod	15	1	15	0.30	0.583
Explant type × Photoperiod	49	2	25	0.49	0.619
ERROR	8689	175	50		

**Table 2:** Percentage of embryogenic calli and the average number of somatic embryos achieved per explant in OMc medium without growth regulators. Different letters correspond to statistically significant differences (*p* ≤ 0.05).

	0 h			16 h		
	Radicle	Proximal	Distal	Radicle	Proximal	Distal
Number of explants in culture	97	259	132	127	135	92
Embryogenic calli (%)	17 b	16 b	12 c	22 a	7 d	16 b
Average number of embryos per calli	1.66 b	1.25 b	1.55 b	2.54 a	1.50 b	1.55 b

**Table 3:** Percentage of embryogenic calli and the average number of somatic embryos achieved in radicles inoculated on ECO medium during two subcultures, 30 days each, and under two light regimes, 16 h and 0 h photoperiod. Different letters correspond to statistically significant differences (*p* ≤ 0.05).

	Subculture I		Subculture II	
	0 h	16 h	0 h	16 h
Number of explants in culture	70	74	75	83
Embryogenic calli (%)	29 d	50 b	44 c	63 a
Average number of embryos per calli	2.7 c	7.5 b	3.95 c	13.6 a
